# Home sweet home: sand flies find a refuge in remote indigenous villages in north-eastern Brazil, where leishmaniasis is endemic

**DOI:** 10.1186/s13071-019-3383-1

**Published:** 2019-03-26

**Authors:** Kamila Gaudêncio da Silva Sales, Débora Elienai de Oliveira Miranda, Pietra Lemos Costa, Fernando José da Silva, Luciana Aguiar Figueredo, Sinval Pinto Brandão-Filho, Filipe Dantas-Torres

**Affiliations:** 1 Department of Immunology, Aggeu Magalhães Institute, Oswaldo Cruz Foundation (Fiocruz), Recife, Brazil; 2 State Secretary of Health of Pernambuco, Recife, Brazil

**Keywords:** Cutaneous leishmaniasis, Dogs, Phlebotomine sand fly, Rural area

## Abstract

**Background:**

From 2012 to 2013, an outbreak of cutaneous leishmaniasis by *Leishmania braziliensis* was detected in indigenous villages located in a remote rural area of Pernambuco state, north-eastern Brazil. Considering that the principal activities of this indigenous community are farming and crop plantation, and also that the outbreak involved many children, we investigated the presence of sand fly vectors inside human houses and also the exposure of dogs to leishmanial parasites. Our general objective was to gather epidemiological data that could indicate the occurrence of a peri-domestic/domestic transmission cycle of *L. braziliensis* in these indigenous villages.

**Methods:**

From March 2015 to March 2016, sand flies were collected using light traps in the indoor and immediate outdoor environments in the three indigenous villages that reported the most cutaneous leishmaniasis cases during the 2012–2013 outbreak. Moreover, samples obtained from 300 dogs living in the outbreak villages and two nearby villages were tested by a rapid immunochromatographic test and by a real-time PCR for detecting anti-*Leishmania* antibodies and *Leishmania* DNA, respectively.

**Results:**

In total, 5640 sand flies belonging to 11 species were identified. Males (*n* = 3540) predominated over females (*n* = 2100). *Migonemyia migonei* (84.3%) was the most abundant species, followed by *Evandromyia lenti* (5.5%), *Lutzomyia longipalpis* (4.1%), *Nyssomyia intermedia* (1.6%) and *Micropygomyia capixaba* (1.4%), representing together ~97% of the sand flies collected. Nine out of the 11 species identified in this study were found indoors, including *M. migonei*, *L. longipalpis* and *N. intermedia*, which are proven vectors of *Leishmania* spp. Out of 300 dogs tested, 26 (8.7%) presented anti-*Leishmania* antibodies and six (2%) were *Leishmania* DNA-positive. The level of exposure in dogs living in the indigenous villages where the 2012–2013 outbreak of human CL was detected was almost 2-fold higher than in the two nearby villages (11.0 *vs* 6.2% for serology and 2.6 *vs* 1.4% for real-time PCR).

**Conclusions:**

The results suggest that different sand fly vectors may be adapted to human dwellings, thus increasing the risk of transmission in the indoor and immediate outdoor environments. The adaptation of sand flies to the indoor environment in the studied indigenous villages may be partly explained by the poor housing conditions and the proximity of the houses to crop plantations and forest fragments.

**Electronic supplementary material:**

The online version of this article (10.1186/s13071-019-3383-1) contains supplementary material, which is available to authorized users.

## Background

Leishmaniases are neglected tropical diseases, which cause significant morbidity and mortality in endemic areas, particularly in tropical and subtropical regions of the world [1]. Brazil, India, Bangladesh, Sudan, South Sudan and Ethiopia account for more than 90% of the global cases of visceral leishmaniasis (VL), with an estimated 200,000 to 400,000 new cases per year [2]. About 75% of the global incidence of cutaneous leishmaniasis (CL) occurs in Afghanistan, Algeria, Colombia, Brazil, Iran, Syria, Ethiopia, Sudan, Costa Rica and Peru, with an estimated 0.7 to 1.2 million new cases per year [2]. From a global perspective, Brazil is one of the main foci of leishmaniases, with an annual average incidence of 1.7 and 8.0 new VL and CL cases per 100,000 population, respectively, during the period 2013–2017 [3].

CL and VL are primarily zoonoses, with wild animals (e.g. forest rodents) and domestic dogs, respectively, being involved as reservoirs in their zoonotic transmission cycles [4, 5]. Outbreaks of zoonotic CL in Brazil are commonly detected among males at working age who enter the forest for various reasons, such as for military training [6]. Deforestation (e.g. for road construction and crop plantation) and population movements from non-endemic to endemic areas (and *vice versa*) are also risk factors for both CL and VL [7]. In recent years, CL and VL have spread across different Brazilian regions, indicating the ineffectiveness of control measures to reduce the burden of the disease in both rural and urban areas [8, 9].

Degradation of natural habitats may force sand fly vectors to adapt to the modified environment [10]. Indeed, blood-feeding insects such as sand flies are commonly attracted to human dwellings, where they may find food sources (e.g. domestic animals and humans) [11], resting places and breeding sites [12]. Certainly, the adaptation of sand fly vectors to human dwellings may increase the risk of *Leishmania* spp. transmission in the peri-domestic and domestic environments.

The increasing interaction between humans, domestic and wild animals, in wild, peri-domestic and domestic environments has caused profound changes in the epidemiology of leishmaniases in the past decades [7]. For instance, CL caused by *Leishmania braziliensis* is a zoonosis maintained by multitude of small mammals (e.g. forest rodents and marsupials) and sand fly vectors, which have adapted to the peri-domestic and domestic environments. In endemic areas, dogs are frequently exposed to sand fly vectors and are often infected by *L. braziliensis* [13]. While dogs play no role as reservoirs of this parasite [14], they can play a useful role as a sentinel host [15].

From 2012 to 2013, an outbreak of CL was detected in indigenous villages located in a remote rural area of Pernambuco state, north-eastern Brazil. These villages are settled in legally-expropriated lands and are presently home to the tribe Xukuru de Ororubá. While many ancient indigenous traditions are maintained by the Xukuru people, their principal working activities are farming and crop plantation for their own subsistence. Bearing this in mind and also considering that the outbreak detected in 2012–2013 involved many children, we investigated the presence of sand fly vectors inside human houses and also the exposure of dogs to leishmanial parasites. Our general objective was to gather epidemiological data that could indicate the occurrence of a peri-domestic/domestic transmission cycle of *L. braziliensis* in these indigenous villages.

## Methods

### Study area

**Fig. 1:**
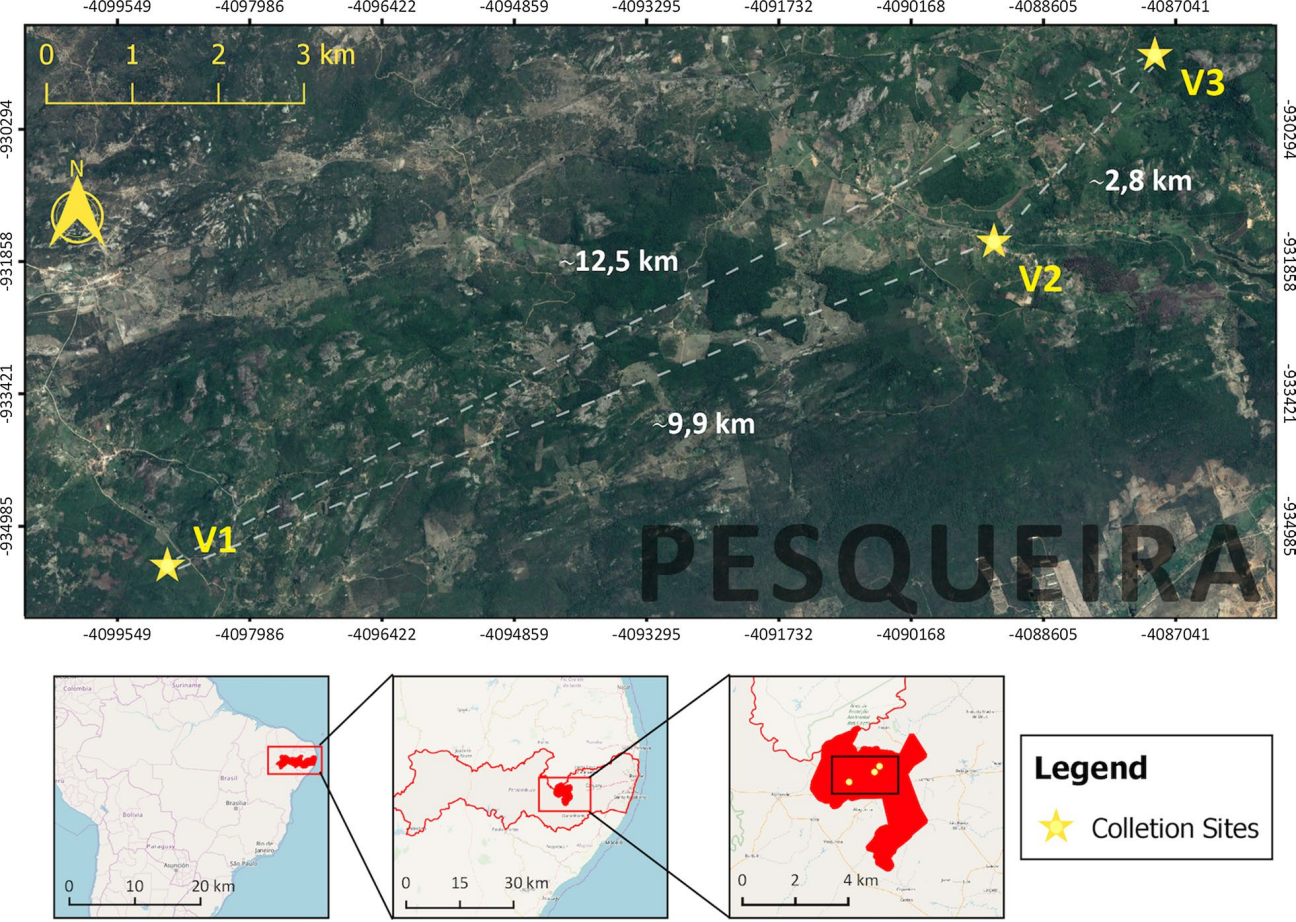
Location of the three indigenous villages (V1, Guarda; V2, Santana; and V3, Afetos) studied herein. Pesqueira, Pernambuco, Brazil. Copyright: Creative Commons Attribution-ShareAlike 3.0 Licence (https://creativecommons.org/licenses/by-sa/3.0/). The map was created using QGIS and publicly available shapefiles from QGIS web site [55]

This study was carried out in the municipality of Pesqueira, which is located in the agreste region of Pernambuco, a narrow zone between the Atlantic forest zone (zona da mata) and the semiarid region (sertão). In particular, three indigenous villages were surveyed in this study: Guarda (V1; 8°21′49.6″S, 36°48′47.1″W, altitude: 844 m above sea level), Santana (V2; 8°20′12.3″S, 36°43′38.1″W, altitude: 850 m) and Afetos (V3; 8°19′06.1″S, 36°42′37.3″W, altitude: 965 m) (Fig. 1). Geographical coordinates and altitude of each village were recorded using a Garmin eTrex Venture HC GPS (Garmin International, Olathe, USA).

These villages are part of the 24 indigenous villages of the tribe Xukuru de Ororubá, which occupy approximately 27,000 hectares of a chain of mountains named Serra do Ororubá. The villages are located in a rural area and the native vegetation is represented by semi-decidual and deciduous forests, although most of the original forest coverage has been substituted by crop plantations [16]. The landscape is represented by a xeric shrubland and thorn forest, which consists primarily of small, thorny trees that shed their leaves seasonally. The ground layer is made up of cacti, thick-stemmed plants, thorny brush and arid-adapted grasses. The climate of this area is semiarid, characterized by low humidity and little rainfall. The raining period ranges from February to July, with an annual average temperature of 26 °C (range, 24–27 °C), average relative humidity of 76% (range, 69–86%) and average precipitation of 700 mm^3^.

The population of the villages is currently around 2720 [17]. The local economy is mostly based on agriculture, with plantations of bananas, beans, cassava, corn and vegetables, as well as dairy cattle and goat farming [18]. Many of the houses are precarious and lack basic sanitation. Children and teenagers typically go to school in the morning and afternoon, respectively. In their spare time they play and are at home early in the evening, particularly young children. Domestic animals (e.g. dogs, cats and chickens) are common both indoors and outdoors of human houses.

### Collection and morphological identification of sand flies

Sand fly collections were carried out monthly, from March 2015 to March 2016 (except in October 2015, for logistic reasons) for two to three consecutive nights. Collection sites (houses) were chosen based on the occurrence of human cases of CL. Some of the houses were made of mud walls and thatched roofs, with obvious openings that may facilitate the entrance of insects during the night. Moreover, some of the houses were surrounded by native vegetation.

Each night, one to four CDC light traps were installed in each village, operating from 18:00 h to 6:00 h, for a total of 253 traps installed and 3036 cumulative hours of trapping. Each trap was positioned 1.5 m above the ground in two types of environments: indoor (living rooms and bedrooms) and outdoor (backyards with chicken coop, goats and/or dogs). All specimens collected were transferred to labelled vials containing 70% ethanol and subsequently identified using morphological keys for American sand flies based on characters of male genitalia, female spermathecae and pharyngeal armature [19]. The nomenclature of sand fly species followed Galati’s proposal [20]. Females collected indoors and outdoors during the last seven months of collection (from August 2015 to March 2016) were classified as engorged (blood in the abdomen, total or partial) or unfed (no visible blood in the abdomen).

### Meteorological data

Monthly average temperature (°C), relative humidity (%) and rainfall (mm) data were obtained from station 82900 of the Technology Institute of Pernambuco (ITEP). The saturation deficit (SD) was calculated as follows: SD = (1 – RH/100) × 4.9463 × *e*^0.0621^ ^× T^, where RH is relative humidity and T is temperature [6].

### Canine blood collection and diagnostic procedures

Dogs living in the investigated indigenous villages (Guarda, Santana and Afetos) and two nearby villages (Cimbres and São José) were chosen as sentinel hosts. From March to June 2015, blood samples were collected (~5 ml) from 300 privately-owned dogs. Aliquots of ~2 ml and ~3 ml were added to EDTA tubes (Greiner Bio-One GmbH, Kremsmünster, Austria) and gel serum separator tubes (Greiner Bio-One GmbH), respectively. Gel serum separator tubes were centrifuged at 2000×*g* for 10 min for serum separation. The obtained sera and blood samples were stored at -20 °C.

Genomic DNA extraction from EDTA-blood samples was performed using the PureLink® Genomic DNA Mini Kit (Invitrogen, Carlsbad, CA, USA), according to the manufacturer’s instructions. The quantity and purity of the extracted DNA were assessed using a NanoDrop 2000c Spectrophotometer (Thermo Fisher Scientific, Waltham, MA, USA). Extracted DNA samples were stored at -20 °C until testing.

Real-time PCR reactions were performed using the primers LEISH-1 (5′-AAC TTT TCT GGT CCT CCG GGT AG-3′) and LEISH-2 (5′-ACC CCC AGT TTC CCG CC-3′) and the TaqMan® probe FAM-5′-AAA AAT GGG TGC AGA AAT-3′-non-fluorescent quencher-MGB, as described elsewhere [21]. The final reaction volume was adjusted to 25 μl containing 5.5 μl of type 1 (ultrapure) water, 2.25 μl of each primer (900 nM), 0.5 μl of probe (200 nM), 12.5 μl of TaqMan® Universal PCR Master Mix (Applied Biosystems, Carlsbad, CA, USA) and 2 μl of DNA template. PCR cycling conditions were 50 °C for 2 min, 95 °C for 10 min, then 40 cycles at 95 °C for 15 s and at 60 °C for 1 min. All samples were tested in duplicate and no template control (NTC) and positive controls (DNA extracted from cultured *L. infantum* promastigotes) were included in each PCR run. Reactions were run on a 7500 Real Time PCR System (Applied Biosystems, Foster City, CA, USA) and the results analysed using the 7500 software v.2.0.5.

Dog sera were tested using DPP LVC (Bio-Manguinhos, Rio de Janeiro, RJ, Brazil), according to the manufacturer’s instructions. This test uses recombinant antigens and it is the official screening test used by public health authorities in Brazil [22]. Results were read after 10 min and interpreted as follows: negative (only control red line present), positive (control and sample red lines present) and invalid (control red line absent).

### Human cases

Secondary data regarding human cases of CL detected during the 2007–2017 in the indigenous villages were obtained from the Brazilian Information System on Diseases of Compulsory Declaration [3]. In particular, we were interested in all CL cases detected in the outbreak of 2012–2013. Variables of interest were year and month of notification, gender, place of residence, and age. Data were obtained and processed anonymously.

### Diversity indices and statistical analyses

Sand fly species richness and diversity were assessed using the following parameters: species richness (*S*), number of individuals (*n*), Shannon’s diversity index (*H*’) and Pielou’s equitability index (*J*’). We also computed the species accumulation curve (sample-based rarefaction) as a function of number of samples using Maoʼs tau, with standard deviation; in the graphical plot, the standard errors were converted to 95% confidence intervals. Diversity indices and species accumulation curve were calculated using PAST, v.3.23 for Mac OS [23].

Before statistical analysis, normality of data was assessed using Lilliefors. Pearson’s (*r*) or Spearman’s (*r*_*s*_) correlation coefficients were used to determine the correlation between the meteorological variables and the relative frequencies of sand flies (i.e. number of individuals per hour of trapping). Student’s t-test was used for comparing the relative frequencies of sand flies collected monthly indoors *vs* outdoors. To compare the abundance of each species indoors *vs* outdoors, we calculated the index of species abundance (ISA), which was then converted to a scale of zero to one, through the standardized index of species abundance (SISA), where the value 1.00 represents the most abundant species [24]. The Kruskal-Wallis H-test (with Dunn’s *post-hoc* test) was used to compare the relative frequencies of sand flies collected monthly in the three surveyed indigenous villages. Chi-square test was used to assess whether positivity to *Leishmania* spp. varied according to dog data including sex (male, female), age (≤ 1 year, > 1 year), clinical status (healthy, sick) and housing condition (domiciled, semi-domiciled). We also used the Chi-square test to determine whether there was a significant difference between the frequencies of engorged and unfed females collected indoors *vs* outdoors. Statistical analyses were performed using BioEstat v.5.3 (Mamirauá Institute of Sustainable Development, Tefé, AM, Brazil) and *P* ≤ 0.05 was considered statistically significant.

## Results

### Sand fly species and numbers

**Fig. 2:**
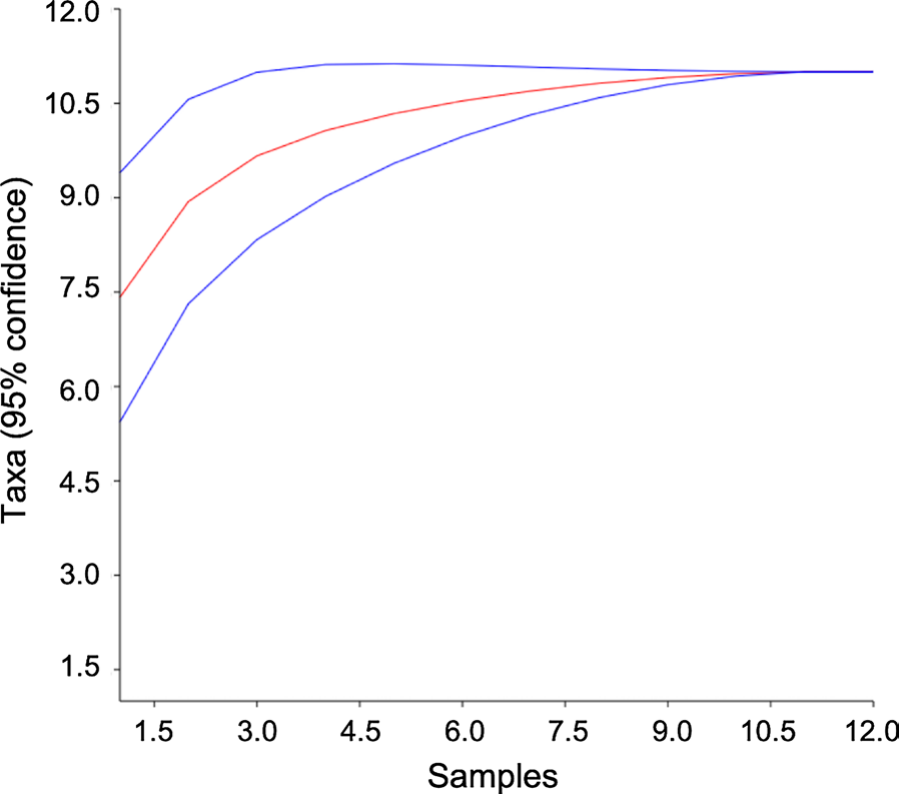
Species accumulation curve (Mao’s tau function) (red line) and 95% confidence interval (blue lines) for sand flies in Pesqueira, Pernambuco, Brazil

A total of 5640 sand flies were collected and morphologically identified (Table 1). The species accumulation curve reached saturation at the 10th sampling event (Fig. 2), with 11 species identified.

**Table 1:** Number (*n*) and percentage (%) of sand flies collected indoors and outdoors in the surveyed indigenous villages (V1–V3) in Pesqueira, Pernambuco, Brazil, from March 2015 to March 2016, according to species and sex. Sex ratio (female:male) is also provided

Species	Indoor							Outdoor						
	Female		Male		Total		Sex ratio	Female		Male		Total		Sex ratio
	*n*	%	*n*	%	*n*	%		*n*	%	*n*	%	*n*	%	
*Micropygomyia capixaba*	46	59.0	3	3.8	49	62.8	15.3	24	30.8	5	6.4	29	37.2	4.8
*Evandromyia evandroi*	5	7.8	1	1.6	6	9.4	5.0	53	82.8	5	7.8	58	90.6	10.6
*Micropygomyia villelai*	4	11.1	10	27.8	14	38.9	0.4	16	44.4	6	16.7	22	61.1	2.7
*Nyssomyia intermedia* ^a^	3	3.4	12	13.5	15	16.9	0.3	34	38.2	40	44.9	74	83.1	0.9
*Evandromyia lenti*	18	5.8	30	9.7	48	15.6	0.6	72	23.4	188	61.0	260	84.4	0.4
*Lutzomyia longipalpis* ^a^	25	10.9	46	20.0	71	30.9	0.5	50	21.7	109	47.4	159	69.1	0.5
*Migonemyia migonei* ^a^	438	9.2	620	13.0	1058	22.3	0.7	1272	26.8	2423	51.0	3695	77.7	0.5
*Evandromyia sallesi*	0	0.0	0	0.0	0	0.0	nc	2	66.7	1	33.3	3	100.0	2.0
*Micropygomyia schreiberi*	0	0.0	0	0.0	0	0.0	nc	3	100.0	0	0.0	3	100.0	nc
*Sciopemyia sordellii*	0	0.0	7	24.1	7	24.1	0.0	9	31.0	13	44.8	22	75.9	0.7
*Micropygomyia trinidadensis*	10	21.3	6	12.8	16	34.0	1.7	16	34.0	15	31.9	31	66.0	1.1
Total	549	42.8	735	57.2	1284	22.8	0.7	1551	35.6	2805	64.4	4356	77.2	0.6

^a^Proven vector species [24]

*Abbreviation*: nc, not calculated

The percentage of females (37.2%, *n* = 2100) was lower than that of males (62.8%, *n* = 3540), with an overall female:male ratio of 0.6:1. A positive correlation between the monthly number of males and females collected during the study (*r*_(10)_ = 0.86, *P* < 0.001) was found. However, the sex ratio varied according to sand fly species, type of environment (indoors *vs* outdoors) (Table 1) and village (Table 2).

**Table 2:** Number (*n*) and percentage (%) of sand flies collected in three indigenous villages (V1**–**V3) in Pesqueira, Pernambuco, Brazil, from March 2015 to March 2016, according to species and sex. Sex ratio (female:male) is also provided

Species	Guarda (V1)							Santana (V2)							Afetos (V3)						
	Female		Male		Total		Sex ratio	Female		Male		Total		Sex ratio	Female		Male		Total		Sex ratio
	*n*	%	*n*	%	*n*	%		*n*	%	*n*	%	*n*	%		*n*	%	*n*	%	*n*	%	
*Micropygomyia capixaba*	31	4.5	7	0.7	38	2.2	4.4	3	1.6	0	0.0	3	0.8	nc	36	3.0	1	0.0	37	1.0	36.0
*Evandromyia evandroi*	32	4.6	2	0.2	34	2.0	16.0	9	4.8	1	0.5	10	2.5	9.0	17	1.4	3	0.1	20	0.6	5.7
*Micropygomyia villelai*	4	0.6	11	1.1	15	0.9	0.4	3	1.6	0	0.0	3	0.8	nc	13	1.1	5	0.2	18	0.5	2.6
*Nyssomyia intermedia* ^a^	10	1.4	14	1.4	24	1.4	0.7	1	0.5	2	1.0	3	0.8	0.5	26	2.1	36	1.5	62	1.7	0.7
*Evandromyia lenti*	50	7.2	125	12.5	175	10.3	0.4	12	6.4	28	13.6	40	10.2	0.4	28	2.3	65	2.8	93	2.6	0.4
*Lutzomyia longipalpis* ^a^	70	10.1	126	12.6	196	11.6	0.6	0	0.0	5	2.4	5	1.3	0.0	5	0.4	24	1.0	29	0.8	0.2
*Migonemyia migonei* ^a^	483	69.8	704	70.5	1187	70.2	0.7	154	82.4	163	79.1	317	80.7	0.9	1073	88.0	2176	93.1	3249	91.4	0.5
*Evandromyia sallesi*	0	0.0	0	0.0	0	0.0	nc	2	1.1	1	0.5	3	0.8	2.0	0	0.0	0	0.0	0	0.0	nc
*Micropygomyia schreiberi*	0	0.0	0	0.0	0	0.0	nc	1	0.5	0	0.0	1	0.3	nc	2	0.2	0	0.0	2	0.1	nc
*Sciopemyia sordellii*	7	1.0	6	0.6	13	0.8	1.2	2	1.1	4	1.9	6	1.5	0.5	0	0.0	10	0.4	10	0.3	0.0
*Micropygomyia trinidadensis*	5	0.7	4	0.4	9	0.5	1.3	0	0.0	2	1.0	2	0.5	0.0	19	1.6	17	0.7	36	1.0	1.1
Total	692	40.9	999	59.1	1691	30.0	0.7	187	47.6	206	52.4	393	7.0	0.9	1219	34.3	2337	65.72	3556	63.0	0.5

^a^Proven vector species [24]

*Abbreviation*: nc, not calculated

**Fig. 3:**
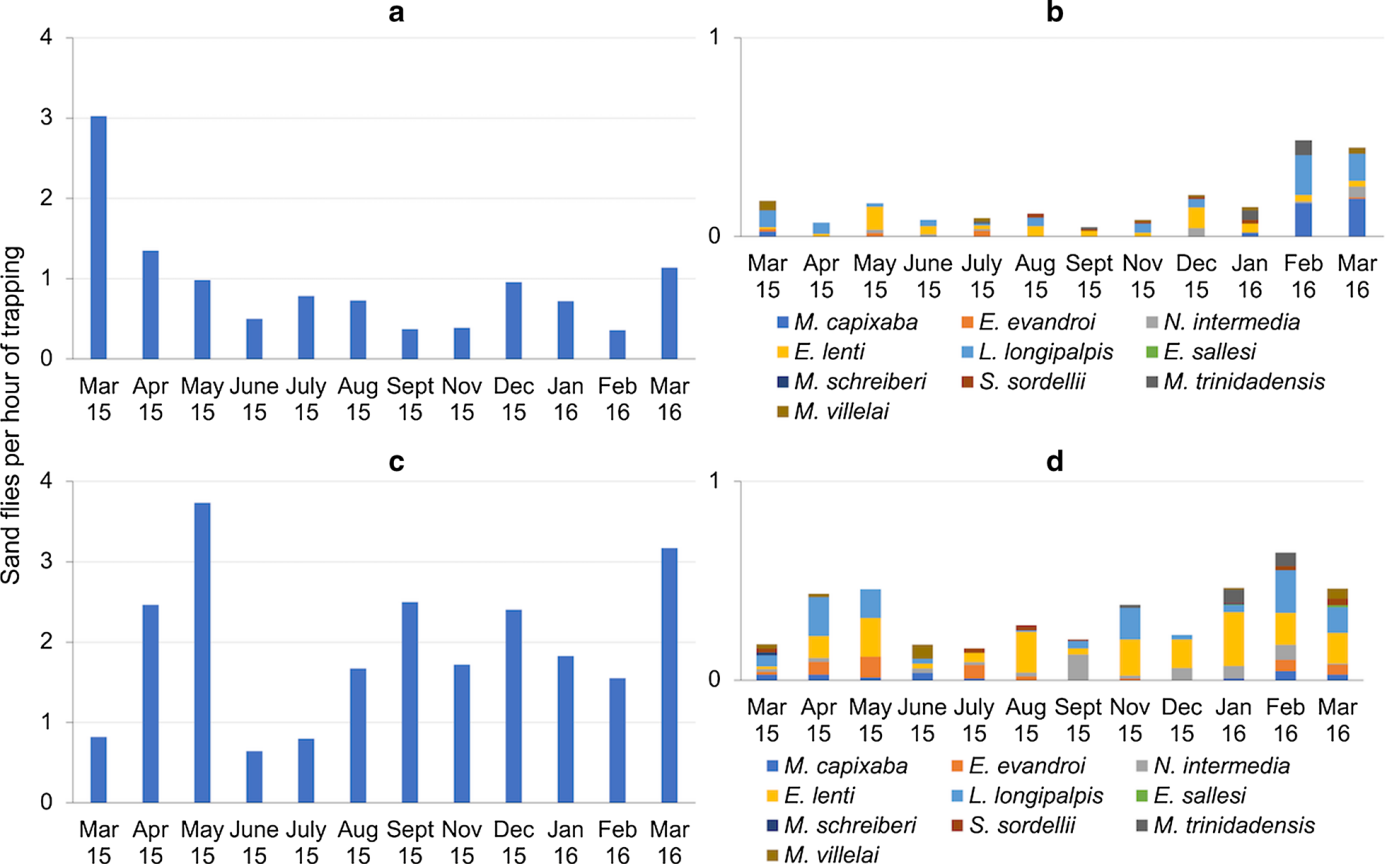
Monthly relative frequency of sand flies (number of individuals per hour of trapping) collected indoors (**a** and **b**) and outdoors (**c** and **d**) according to species (**a** and **c** *Migonemyia migonei*; **b** and **d** remaining species), Pesqueira, Pernambuco, Brazil

Most sand flies were collected outdoors (77.2%; *n* = 4356; mean = 2.36 sand flies per hour of trapping) as compared to indoors (22.8%; *n* = 1284; mean = 1.08 sand flies per hour of trapping) (Student’s t-test, *t*_(22)_ = 3.14, *P* = 0.005). However, 9 out of 11 species found in this study were collected inside the houses. Some sand fly species (i.e. *Evandromyia lenti*, *Migonemyia migonei* and *Lutzomyia longipalpis*) were consistently collected indoors throughout the entire study period (Fig. 3, Additional file 1: Table S1). According to data recorded from August 2015 to March 2016, 28 out of 232 (12.1%) *M. migonei* females collected indoors and 98 out of 890 (11.0%) those collected outdoors were engorged (*χ*^2^ = 0.21, *df* = 1, *P* = 0.650). Additionally, 2 out of 32 (6.3%) *Nyssomyia intermedia* females collected outdoors were also engorged. All other females belonging to other species, which were collected in the aforementioned period, were unfed.

*Migonemyia migonei* was the most abundant species both indoors (SISA = 1.00) and outdoors (SISA = 1.00). In the outdoor environment, *L. longipalpis* (SISA = 0.90) was the second most abundant species, followed by *E. lenti* (SISA = 0.67), *Evandromyia evandroi* (SISA = 0.67), *N. intermedia* (SISA = 0.58), *Sciopemyia sordellii* (SISA = 0.43), and other less abundant species (Additional file 2: Figure S1). In the indoor environment, *Micropygomyia capixaba* (SISA = 0.81) was the second most abundant species, followed by *E. lenti* (SISA = 0.78), *Micropygomyia trinidadensis* (SISA = 0.54), *L. longipalpis* (SISA = 0.48), *Micropygomyia villelai* (SISA = 0.46), and other less abundant species (Additional file 2: Figure S1).

Among the species collected, *M. migonei* (84.3%), *E. lenti* (5.5%), *L. longipalpis* (4.1%), *N. intermedia* (1.6%) and *M. capixaba* (1.4%) were the most frequent, representing together ~97% of the total sand flies captured. These three species were consistently trapped during the whole study period and in the three studied villages. However, *M. migonei* specimens were more frequently collected in V3, whereas most *E*. *lenti* and *L*. *longipalpis* in V1. *Evandromyia sallesi* (*n* = 3) and *Micropygomyia schreiberi* (*n* = 3) were the least representative species, being found only in V2 and V3 (Table 2).

The relative frequencies of sand flies collected monthly varied according to village (Kruskal-Wallis H-test, *H* = 18.24, *df* = 2, *P* < 0.001; Dunn’s *post-hoc* test, *P* < 0.05 for V1 *vs* V2 and V2 *vs* V3, and *P* > 0.05 for V1 *vs* V3). The highest number of sand flies was recorded in village V3 (63.0%; *n* = 3556; mean = 2.77 sand flies per hour of trapping), followed by V1 (30.0%; *n* = 1691; mean = 1.68 sand flies per hour of trapping) and V2 (7.0%; *n* = 393; mean = 0.53 sand flies per hour of trapping). The highest species richness was found in V2, where all 11 species found in this study were present (Table 3). The species diversity and equitability were higher in V1 (*H’* = 1.06, *J’* = 0.48) and V2 (*H’* = 0.81, *J’* = 0.34), corresponding to the villages with lower altitude (i.e. 844 and 850 m, respectively).

**Table 3:** Diversity indices in three indigenous villages in Pesqueira, Pernambuco, Brazil, from March 2015 to March 2016

Index	Guarda (V1)	Santana (V2)	Afetos (V3)
Species richness	9	11	10
Individuals (*n*)	1691	393	3556
Shannon (*H*’)	1.06	0.81	0.46
Equitability (*J*’)	0.48	0.34	0.20

**Fig. 4:**
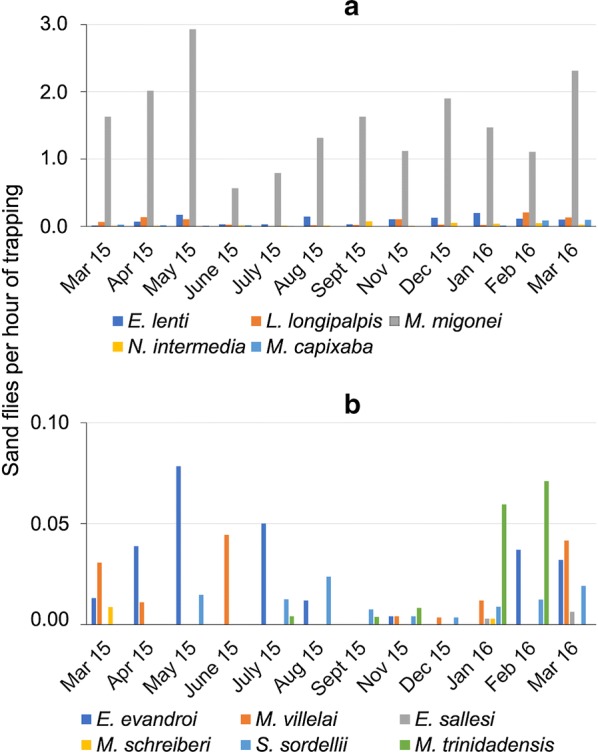
Monthly relative frequency of sand flies (number of individuals per hour of trapping) collected in Pesqueira, Pernambuco, Brazil (**a** the five most common species; **b** the remaining species)

**Fig. 5:**
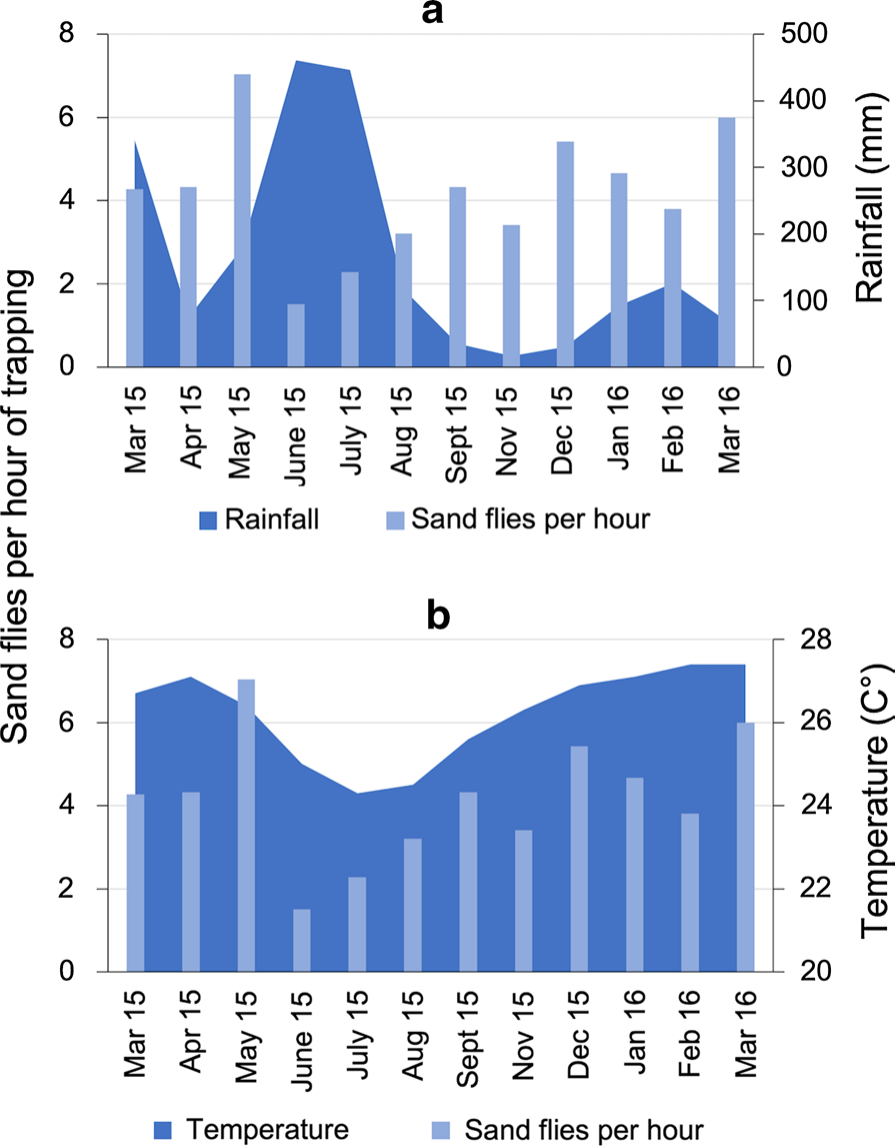
Monthly relative frequency of sand flies (number of individuals per hour of trapping) in relation to rainfall (**a**) and temperature (**b**) in Pesqueira, Pernambuco, Brazil

The months with the greatest number of sand flies collected were May 2015 and March 2016 (Fig. 4), when the monthly average precipitations were 179 and 69.2 mm, monthly average temperatures 26.4 and 27.4 °C and monthly average relative humidity 77.3 and 74%, respectively (Fig. 5).

The months with higher temperatures and lower relative humidity coincided with peaks in sand fly population. Indeed, the relative frequency of sand flies (i.e. sand flies per hour of trapping) collected monthly was positively correlated with temperature (*r*_(10)_ = 0.62, *P* = 0.033). On the other hand, no significant correlation was found between the relative frequency of sand flies with relative humidity (*r*_(10)_ = -0.44, *P* = 0.152), precipitation (*r*_*s*_ = -0.32, *P* = 0.308) or saturation deficit (*r*_(10)_ = 0.49, *P* = 0.107). The most abundant species collected (i.e. *M. migonei*, *E. lenti* and *L. longipalpis*) were collected mainly in months with an average relative humidity < 75%.

### Canine exposure to *Leishmania* spp

Out of 300 dogs tested, 26 (8.7%) were positive by serology and six (2%) by real-time PCR. Six dogs were simultaneously positive for both the tests. The highest positivity rates for both serology and real-time PCR were recorded in the outbreak villages (serology = 11.0%, 17/155; real-time PCR = 2.6%, 4/155), as compared with nearby villages (serology = 6.2%, 9/145; real-time PCR = 1.4%, 2/145). Most of the dogs presented apparent clinical signs (*n* = 179) and were semi-domiciled (*n* = 265). No significant association was found between positivity (serology and/or real-time PCR) and variables such as the sex (*χ*^2^ = 1.11, *df* = 1, *P* = 0.293), age (*χ*^2^ = 2.78, *df* = 1, *P* = 0.096), clinical status (*χ*^2^ = 3.52, *df* = 1; *P* = 0.061) and housing condition (*χ*^2^ = 1.69, *df* = 1, *P* = 0.194).

### Human cases

From 2007 to 2017, 49 human cases of CL were notified in 12 indigenous villages of Pesqueira. Of these, 40 cases were diagnosed from February 2012 to December 2013, with 77.5% (*n* = 31) of the cases being diagnosed in 2012. During this outbreak, 67.5% of the cases were detected in V1 (50%, *n* = 20), V2 (12.5%, *n* = 5) and V3 (5%, *n* = 2). Cases were notified during almost all months of the year, except in May and June. The months with more cases recorded during outbreak were: March 2012 (*n* = 7) and October 2012 (*n* = 14). Men (*n* = 29) were more frequently affected than women (*n* = 11), and most cases (62.5%) were from 3 to 10 years-old (25%) and 11 to 17 years-old (37.5%), with age ranging from 3 to 66 years.

## Discussion

Throughout the study period, we identified 11 sand fly species in the investigated indigenous villages. *Migonemyia migonei*, *E. lenti*, *L. longipalpis*, *N. intermedia* and *M. capixaba* were the most abundant species, present in all villages, both indoors and outdoors. From an epidemiological point of view, this finding is very important because it may suggest a year-long risk for the transmission of *Leishmania infantum* and *L. braziliensis* in this region. *Lutzomyia longipalpis* and *M*. *migonei* are incriminated as vectors of *L. infantum* and *L. braziliensis*, respectively, in Brazil and other Latin American countries [25]. In addition, *M. migonei* has been strongly suggested as a vector of *L. infantum* in some foci [26, 27] and a recent laboratory study reinforced this hypothesis [28]. Furthermore, *N. intermedia* is also a proven vector of *L. braziliensis* in Brazil [25]. Considering the presence of potential vectors during the whole year, further research focused on detecting DNA (by PCR) or promastigotes (by dissection and microscopical examination) of *Leishmania* spp. in sand flies could provide valuable data on the transmission pattern in this area, which may include the participation of multiple vectors.

Our results support our initial hypothesis that sand flies may be adapted to human dwellings in the studied indigenous villages. For instance, *M. capixaba* is a sylvatic species, generally found in forests and marginal areas [29]. However, in the present study, this species was the second most abundant species indoors (SISA = 0.81), after *M. migonei* (SISA = 1.00). Notably, 46 out of 49 *M. capixaba* specimens caught indoors were female, which could suggest an endophilic behaviour, although none of them were engorged. Future studies, with a larger number of specimens, are needed to assess the blood meals of *M. capixaba* females collected indoors and outdoors in these villages. The houses where *M. capixaba* were found indoors have openings, as did most of the houses in the studied indigenous villages. Moreover, some of the houses were surrounded by native vegetation, which may have favoured the encounter of *M. capixaba* in their interior. A study carried out in São Vicente Férrer (agreste region of Pernambuco) [30] reported a single female in the peridomicile and 24 males and 31 females in the forest environment. In Caruaru (agreste region of Pernambuco) [31] reported only three females of *M. capixaba* in the intradomicile. These findings suggest that sylvatic sand fly species (e.g. *M. capixaba*) may find a home inside human houses in the studied indigenous villages. Indeed, also sand flies incriminated as vectors of *Leishmania* spp. (i.e. *M. migonei*, *N. intermedia* and *L. longipalpis*) were consistently collected inside the investigated houses during this study. Moreover, 12.1% of *M. migonei* females caught indoors contained fresh blood in their abdomen, suggesting an endophilic behaviour. Overall, these findings may indicate a constant, close contact between sand fly vectors, domestic animals and humans, potentially increasing the risk of *Leishmania* spp. transmission. Several factors may drive the adaptation of sand flies to human dwellings, including deforestation, construction of houses close to forest fragments, poor housing conditions and presence of animal sheds in the backyards. All these factors were observed in the indigenous villages surveyed in this study.

Similar studies conducted in Pernambuco reported a species richness ranging between 4–25 species [6, 27, 30–32]. Until this study, 41 sand fly species were considered to be present in Pernambuco [29]. With the record of *M. trinidadensis*, this study increases the number of sand fly species of Pernambuco to 42, corresponding to approximately 4.3 species per 10,000 km^2^. Pernambuco has a rich sand fly fauna as compared with other Brazilian states [29], such as Alagoas (3.2 per 10,000 km^2^) and São Paulo (3.1 per 10,000 km^2^). Incidentally, some authors have mentioned the presence of *M. trinidadensis* in Pernambuco [33], but provided no evidence or reference supporting this statement. Indeed, this species was not considered in subsequent sand fly species checklists of this state [13, 29].

The distribution of CL appears to be influenced by altitude. We observed a high species diversity and lower species dominance in the villages with altitude ~850 m (V1 and V2), where 62.5% of the CL cases reported from 2007 to 2017 were concentrated. A study conducted in south-eastern Brazil showed that the number of CL cases decreased progressively with altitude [34]; most cases occurred at 650–750 m and no case occurred at 850–950 m. This is in partial agreement with our results, since CL cases were detected in V3, which is located at an altitude of 965 m. It is worth noting that the highest number of the potential vectors *M*. *migonei* and *N. intermedia* were found exactly in V3, where only two CL cases were reported in ten years. This suggests that the risk of CL in this area may not be directly correlated with sand fly abundance.

The overall number of males was higher than females, as reported in different studies conducted in other regions of Brazil [35–38]. It is acknowledged that the sex ratio may be influenced by trapping methods, with light traps usually attracting more males than females [39]. However, the sex ratio varied widely according to species and environment (indoors *vs* outdoors, e.g. *M. villelai*), being close to unity in some (e.g. *M. trinidadensis*) and female-biased in others (e.g. *M. capixaba* and *E. evandroi*). This indicates that females of some species may be more phototropic than others, as emphasized elsewhere [39].

The highest sand fly population peaks occurred in May 2015 and March 2016, corresponding to the pre-rainy season in 2015 and following the first rains in February 2016, respectively. The number of sand flies collected was positively correlated with temperature. It means that sand flies were more frequently trapped during hot months. It is worth noting that in the agreste region of Pernambuco the rains are unevenly distributed throughout the year, occurring mainly from February to July. This factor may be an important driver of the seasonality of sand flies in this region, similarly to what occurs in the semiarid region of Ceará state [40]. The decline in the sand fly collections during torrential rains (June and July) may also be attributed to inherent difficulties in collecting sand flies using light traps under the rain [6]. In other words, the lower trapping success during this period probably related to a reduced flying activity of sand flies during the raining nights rather than due to their absence. In a study conducted in Passira, another municipality located in the agreste region of Pernambuco, the authors reported that 82.4% of the *L*. *longipalpis* specimens were collected in months with relative humidity surpassing 75% [31]. In the present study, most *L*. *longipalpis* specimens (79.1%) were collected in months with a relative humidity less than 75%. These divergent results reinforce the hypothesis that *L. longipalpis* in north-eastern Brazil is less dependent on climate [31] or that the relationship between climate and *L. longipalpis* population may vary locally.

It is worth noting that 50% of the CL cases reported during the outbreak from 2012 to 2013 in Pesqueira were concentrated at V1. Interestingly, most *M. migonei* females (50.2%) collected indoors in this study were collected in V1. While this species displays a sylvatic behaviour in some Brazilian regions, it is recognized that in north-eastern Brazil this species is adapted to different environments, including forest fragments, animal sheds and human houses [41, 42].

In general, most human cases of CL diagnosed in rural and/or forested areas in north-eastern Brazil are males involved in occupational activities that increase their exposure to sand flies [6, 43–45]. In rural settings where children are frequently affected by CL, the transmission cycle of *L. braziliensis* is probably taking place in the indoor or immediate outdoor environments [46], where domestic dogs are also frequently exposed. In the present study, a relatively low (8.7%) overall level of exposure to *Leishmania* spp. infection was detected among dogs living in the studied indigenous villages and two nearby villages, as compared to other studies conducted in Pernambuco [47–50] or elsewhere in Brazil [51, 52]. While the overall level of exposure to *Leishmania* spp. infection in dogs was relatively low, data from notified CL cases and from our serological survey suggest that transmission is taking place in the peri-domestic and/or domestic environments. Indeed, these dogs are typically free roaming during the day, but stay around or inside the houses during the night. Furthermore, considering that remarkably anthropophilic vector species (e.g. *N. intermedia*) [10] were found indoors, the risk of exposure to *L. braziliensis* may be ever higher in humans as compared to dogs. This could partially explain the 2012–2013 outbreak in humans and the relatively low exposure to leishmanial parasites in dogs. Nevertheless, the level of exposure in dogs living in the indigenous villages where the 2012–2013 outbreak of human CL was detected was almost 2-fold higher than in the two nearby villages (11.0 *vs* 6.2% for serology and 2.6 *vs* 1.4% for real-time PCR).

While this was not our primary objective for screening dogs, the low exposure to leishmanial parasites in dogs suggests that these animals are not playing any role as reservoirs of *L. braziliensis* in the study area, which is in line with the current notion that dogs are mere accidental hosts of this parasite [14]. Indeed, previous studies conducted in other endemic foci in Pernambuco have indicated sylvatic and synanthropic rodents as the reservoirs of *L. braziliensis* [4, 53], a hypothesis also supported by an experimental study [54].

## Conclusions

In conclusion, we confirm that proven sand fly vectors are present in the indoor and immediate outdoor environments in indigenous villages where CL is endemic. The adaptation of sand flies to the indoor environment may be related to the poor housing conditions observed in these villages and the proximity of houses to green areas (e.g. crop plantations and forest fragments).

## Additional files

**Additional file 1: Table S1.** Number of sand flies collected indoors and outdoors in each village (V1–V3) in Pesqueira, Pernambuco, Brazil, from March 2015 to March 2016.

**Additional file 2: Figure S1.** Standardized index of species abundance (SISA) of sand fly species collected indoors and outdoors in Pesqueira, Pernambuco, Brazil, from March 2015 to March 2016.
