# Supplementary material for: Home sweet home: sand flies find a refuge in remote indigenous villages in north-eastern Brazil, where leishmaniasis is endemic
Source: Parasit Vectors. 2019 Mar 26;12:118. doi: 10.1186/s13071-019-3383-1 (PMC6434633; doi:10.1186/s13071-019-3383-1)
Supplement: Supplementary file 1 — Additional file 1: Table S1. Number of sand flies collected indoors and outdoors in each village (V1–V3) in Pesqueira, Pernambuco, Brazil, from March 2015 to March 2016. [file 13071_2019_3383_MOESM1_ESM.docx]

**Table S1** Number of sand flies collected indoor and outdoor in each village (V1–V3) in Pesqueira. Pernambuco, Brazil, from March 2015 to March 2016.

| Month/Year | Guarda (V1) | | | | Santana (V2) | | | | Afetos (V3) | | | | Total | | | |
| --- | --- | --- | --- | --- | --- | --- | --- | --- | --- | --- | --- | --- | --- | --- | --- | --- |
|  | Indoor | | Outdoor | | Indoor | | Outdoor | | Indoor | | Outdoor | | Indoor | | Outdoor | |
|  | *n* | % | *n* | % | *n* | % | *n* | % | *n* | % | *n* | % | *n* | % | *n* | % |
| March/2015 | 261 | 37.4 | 52 | 5.2 | 0 | 0.0 | 5 | 1.4 | 8 | 1.5 | 84 | 2.8 | 269 | 21.0 | 141 | 3.2 |
| April/2015 | 90 | 12.9 | 218 | 22.0 | 0 | 0.0 | 7 | 2.0 | 12 | 2.2 | 88 | 2.9 | 102 | 7.9 | 313 | 7.2 |
| May/2015 | 57 | 8.2 | 103 | 10.4 | 4 | 8.9 | 66 | 19.0 | 8 | 1.5 | 438 | 14.5 | 69 | 5.4 | 607 | 13.9 |
| June/2015 | 16 | 2.3 | 27 | 2.7 | 0 | 0.0 | 10 | 2.9 | 42 | 7.8 | 32 | 1.1 | 58 | 4.5 | 69 | 1.6 |
| July/2015 | 19 | 2.7 | 17 | 1.7 | 9 | 20.0 | 26 | 7.5 | 65 | 12.0 | 83 | 2.8 | 93 | 7.2 | 126 | 2.9 |
| August/2015 | 33 | 4.7 | 108 | 10.9 | 11 | 24.4 | 67 | 19.3 | 37 | 6.8 | 129 | 4.3 | 81 | 6.3 | 304 | 7.0 |
| September/2015 | 2 | 0.3 | 61 | 6.1 | 13 | 28.9 | 71 | 20.4 | 30 | 5.5 | 290 | 9.6 | 45 | 3.5 | 422 | 9.7 |
| November/2015 | 16 | 2.3 | 40 | 4.0 | 2 | 4.4 | 15 | 4.3 | 33 | 6.1 | 222 | 7.4 | 51 | 4.0 | 277 | 6.4 |
| December/2015 | 19 | 2.7 | 19 | 1.9 | 0 | 0.0 | 12 | 3.4 | 93 | 17.2 | 443 | 14.7 | 112 | 8.7 | 474 | 10.9 |
| January/2016 | 62 | 8.9 | 86 | 8.7 | 3 | 6.7 | 17 | 4.9 | 29 | 5.4 | 419 | 13.9 | 94 | 7.3 | 522 | 12.0 |
| February/2016 | 57 | 8.2 | 122 | 12.3 | 1 | 2.2 | 19 | 5.5 | 43 | 7.9 | 306 | 10.1 | 101 | 7.9 | 447 | 10.3 |
| March/2016 | 66 | 9.5 | 140 | 14.1 | 2 | 4.4 | 33 | 9.5 | 141 | 26.1 | 481 | 16.0 | 209 | 16.3 | 654 | 15.0 |
| Total | 698 | 100.0 | 993 | 100.0 | 45 | 100.0 | 348 | 100.0 | 541 | 100.0 | 3,015 | 100.0 | 1,284 | 100.0 | 4,356 | 100.0 |
