# Supplementary figures and images for: Home sweet home: sand flies find a refuge in remote indigenous villages in north-eastern Brazil, where leishmaniasis is endemic
Source: Parasit Vectors. 2019 Mar 26;12:118. doi: 10.1186/s13071-019-3383-1 (PMC6434633; doi:10.1186/s13071-019-3383-1)

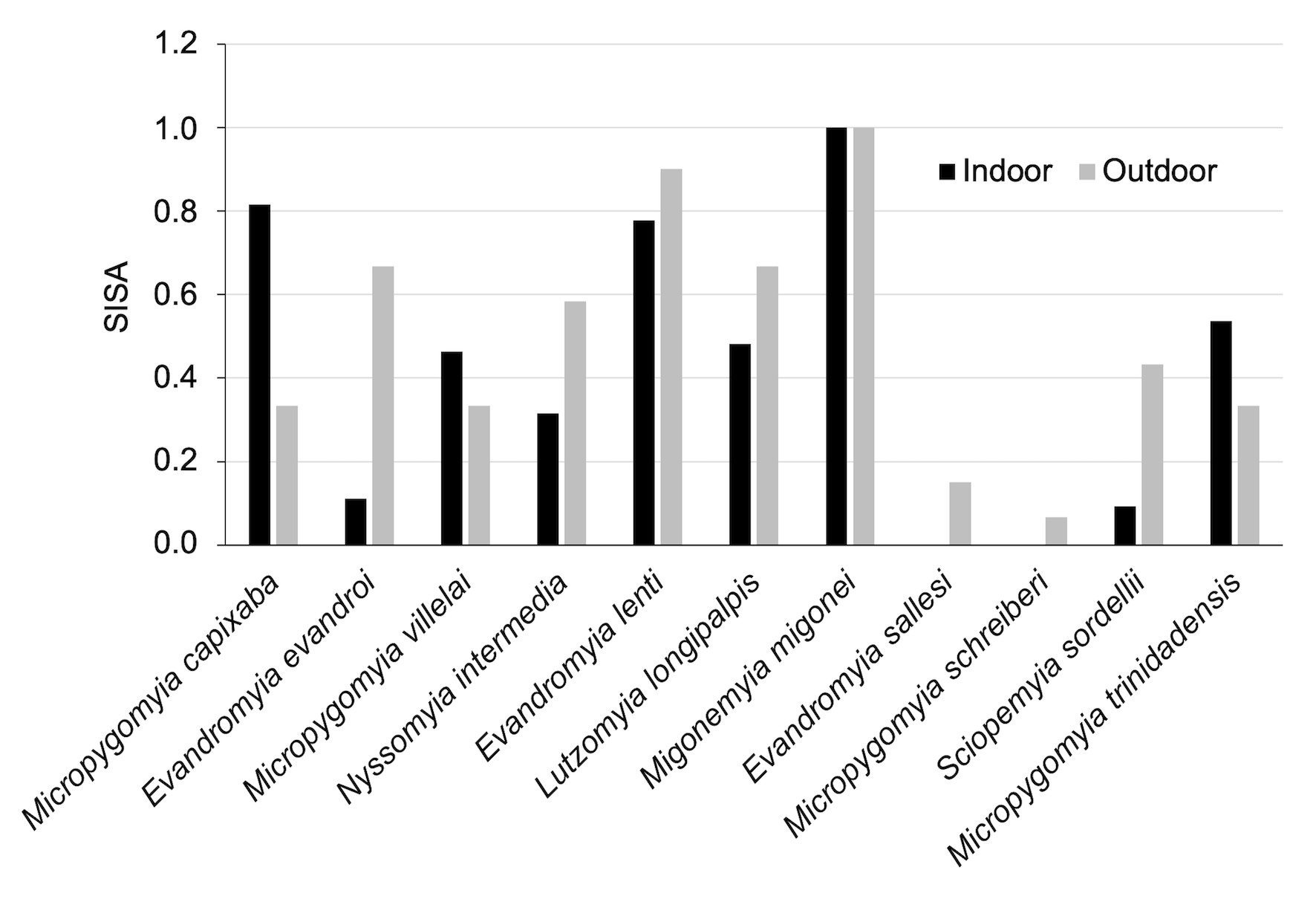

Supplement: Supplementary file 2 — Additional file 2: Figure S1. Standardized index of species abundance (SISA) of sand fly species collected indoors and outdoors in Pesqueira, Pernambuco, Brazil, from March 2015 to March 2016. [file 13071_2019_3383_MOESM2_ESM.tiff]
